# Preventing Long-Term Risk of Obesity for Two Generations: Prenatal Physical Activity Is Part of the Puzzle

**DOI:** 10.1155/2012/470247

**Published:** 2012-12-17

**Authors:** Stephanie-May Ruchat, Michelle F. Mottola

**Affiliations:** ^1^R. Samuel McLaughlin Foundation Exercise and Pregnancy Laboratory, School of Kinesiology, The University of Western Ontario, London, ON, Canada N6A 3K7; ^2^Department of Anatomy and Cell Biology, The University of Western Ontario, London, ON, Canada N6A 3K7; ^3^Children's Health Research Institute, The University of Western Ontario, London, ON, Canada N6A 3K7

## Abstract

*Background*. The period surrounding pregnancy has been identified as a risk period for overweight/obesity in both mother and child because of excessive gestational weight gain (GWG). The promotion of a healthy GWG is therefore of paramount importance in the context of the prevention of obesity in the current and next generations. *Objective*. To provide a comprehensive overview of the effect of prenatal physical activity interventions, alone or in combination with nutritional counselling, on GWG and to address whether preventing excessive GWG decreases the incidence of infant high birth weight and/or postpartum weight retention. *Method*. A search of the PubMed database was conducted to identify all relevant studies. Nineteen studies were included in this review: 13 interventions combining physical activity, nutrition, and GWG counselling and 6 interventions including physical activity alone. *Results*. Prenatal lifestyle interventions promoting healthy eating and physical activity habits appear to be the most effective approach to prevent excessive GWG. Achievement of appropriate GWG may also decrease the incidence of high infant birth weight and postpartum weight retention. *Conclusion*. Healthy eating habits during pregnancy, combined with an active lifestyle, may be important elements in the prevention of long-term risk of obesity for two generations.

## 1. Introduction

There has been a significant increase over the past few decades in the prevalence of maternal overweight (body mass index, BMI ≥ 25 kg/m^2^) and obesity (BMI ≥ 30 kg/m^2^). In the United States, data from the “Pregnancy Risk Assessment Monitoring System” in nine states indicated that pre-pregnancy obesity increased from 13% to 22% between 1993 and 2002 [1]. Worldwide population estimates of pre-pregnancy overweight is approximately 34% [2, 3] and that of pre-pregnancy obesity is 25% [4]. Women who enter pregnancy either normal weight or overweight/obese are at an increased risk of developing obesity or increasing BMI categories because of excessive gestational weight gain (GWG). 

### 1.1. Excessive Gestational Weight Gain: A Link with Obesity Risk in Mother and Child

Excessive GWG has been associated with short- and long-term adverse maternal and infant outcomes [5]. Large weight gain has been linked to postpartum weight retention, which in turn has been associated with long-term risk of maternal obesity [6–11]. Excessive GWG has also been associated with adverse infant outcomes, such as an increased risk of being heavier and fatter at birth [8, 12–15], which increases the infant's risk of becoming overweight/obese later in life [8, 13, 16, 17]. These findings suggest that influences occurring very early in life are contributing to the obesity onset. The promotion of healthy weight gain during pregnancy is therefore of paramount importance to reduce the risk of long-term obesity and associated comorbidities in both the mother and infant. 

### 1.2. Gestational Weight Gain Guidelines

To optimize both maternal and infant outcomes, recommendations regarding healthy weight gain have been developed. The Institute of Medicine (IOM) published in 2009 a revision of the 1990 guidelines for how much weight a woman should gain during pregnancy [5] (Table 1). Using the 1990 IOM recommendations, it has been reported that up to 40% of normal weight women and 60% of overweight/obese women gained excessive weight during pregnancy [11, 13, 18]. However, a study that quantified how the 2009 revisions of the 1990 IOM GWG guidelines changed BMI-specific GWG adherence categories showed that 17.1% of 1990 appropriate weight gaining women would now be classified as excessive gainers, given the new guidelines [19]. Health care providers must therefore determine the women's pre-pregnancy BMI and provide BMI-specific GWG recommendations in accordance with current guidelines, before pregnancy if possible. Further, the importance of healthy weight gain and healthy lifestyle habits to optimize pregnancy outcomes should also be emphasized throughout pregnancy.

### 1.3. Physical Activity during Pregnancy

Research over the past 25 years has demonstrated that healthy women with low-risk pregnancies can safely participate in physical activities without affecting fetal growth and development [20–23]. In fact, maternal physical activity has been identified as an important component of a healthy pregnancy and is beneficial to the mother and fetus [24, 25]. A sedentary lifestyle, on the other hand, may put the mother and fetus at risk for diseases through altered maternal pregnancy adaptations, such as excessive GWG, gestational diabetes, or gestational hypertension [24, 25]. Observational studies showed that physical activity during pregnancy reduces the risk of glucose intolerance, gestational diabetes [26–29], and excessive GWG [30, 31]. 

Active promotion of physical activity for pregnant women is strongly recommended in the absence of either medical or obstetric contraindications by professional organizations [23, 32, 33]. The joint Society of Obstetricians and Gynaecologists of Canada (SOGC) and Canadian Society for Exercise Physiology (CSEP) Clinical Practice Guidelines provide detailed recommendations about the frequency, intensity, time, and type of exercise, following the FITT principle for exercise prescription [23]. Low risk medically pre-screened pregnant women should exercise 3 to 4 times per week, starting with 15 minutes of aerobic activity at a specific target heart rate intensity based on age and increase time slowly to a maximum of 30 minutes per exercise session. All aerobic activity should be preceded by 10 to 15 minutes of warm-up and followed by 10 to 15 minutes of cool-down. Appropriate exercise intensity may be monitored by using target heart-rate zones, the Borg-scale (rating of perceived exertion, RPE), or the “talk test” [23]. Heart-rate zones that are provided in the guidelines correspond to moderate-intensity exercise (i.e., 60–80% of maximal aerobic capacity, VO_2max⁡_) [23].

The recent opinion statement from the SOGC [34] on obesity during pregnancy strongly suggests that regular exercise during pregnancy may help to reduce the risk of medical complications associated with maternal obesity. Overweight and obese women can participate in exercise, if they have no contraindications to being physically active. They should therefore be medically pre-screened and consult their health care provider before engaging in an exercise program. Target heart rate zones developed for normal weight pregnant women [23] may be too difficult for overweight and obese women to obtain. Target heart rate zones for these women were thus developed and validated at a much lower intensity but high enough to achieve aerobic benefits [35]. The American College of Sports Medicine (ACSM) suggested that previously sedentary overweight and obese pregnant women should initiate an aerobic exercise program at an intensity equivalent to 20% to 39% of reserve aerobic capacity (VO_2 reserve_) [36]. The developed and validated target heart rate zones based on age are 102 to 124 beats per minute (bpm) for overweight and obese women 20 to 29 years of age and 101 to 120 bpm for those aged 30 to 39 years [35]. 

Although maternal physical activity has clear health benefits on pregnancy outcomes and is strongly recommended by professional organizations, a large proportion of women do not meet the recommendations for physical activity during pregnancy, based on information collected by interviews or questionnaires [37–40]. Similarly, studies using pedometers to assess the level of physical activity during pregnancy found that many women have sedentary to low physical activity levels during pregnancy and that the levels of activity declined as their pregnancy progressed [41, 42]. Previous studies conducted in our laboratory showed that overweight and obese pregnant women are mostly inactive, accumulating 6,100 ± 1,700 steps per day [43, 44]. However, when a 40-min structured walk (4,300 ± 680 steps per 40-min session) was added to their usual daily activities, these women were taking 10,000 steps or more [43, 44]. As walking is the most reported activity during pregnancy [37–39, 45], providing step-count goals may encourage pregnant women to be more active. The recommendation would be to add 3,000–4,000 steps in a 30–40-min walk (moderate-intensity) to the usual number of daily steps.

Taken together, these findings showed that a large proportion of women are physically inactive during pregnancy. This may be contributing to excessive GWG, high infant weight at birth, and long-term obesity risk in both the mother and child. Pregnancy may be a teachable time when women are motivated to adopt healthy behaviors and may therefore be the perfect opportunity to introduce lifestyle modification strategies promoting physical activity and healthy eating habits in order to achieve a healthy weight gain and pregnancy outcomes. 

Several systematic reviews and meta-analyses assessing the effect of prenatal lifestyle interventions on GWG have been published in 2010–2012 [46–54] and their conclusions were that prenatal lifestyle interventions had a modest effect on the decrease in GWG. However, these papers included only randomised control trials, considered mean GWG as the main outcome, discussed only briefly the adherence to the IOM GWG guidelines and if so, did not differentiate findings between the 1990 and 2009 IOM recommendations. Furthermore, few of these reviews discuss the effect that prenatal interventions may have on the prevention of infant high birth weight and none on the prevention of maternal weight retention after delivery, which we believe to be extremely important in the context of the prevention of obesity in the current and next generations.

 The objective of this integrative review was therefore to provide a comprehensive overview of prenatal physical activity interventions, alone or in combination with a nutritional intervention, whose main objective was to decrease mean GWG and/or prevent excessive GWG and to raise methodological issues and relevant points to discuss in order to highlight the effective/missing aspects of the reviewed interventions. Further, our objective was also to address important issues, such as whether preventing excessive GWG decreases the incidence of infant high birth weight, or prevents postpartum weight retention. Searches of the PubMed database were performed using the following keywords: “exercise" OR “physical activity" OR “intervention" OR “lifestyle" AND “pregnant" AND “weight gain". Relevant references in the literature obtained were further reviewed to identify additional studies on this topic. All types of intervention (randomised controlled trials, historical cohort, single-arm study) published in English were considered. Studies including gestational diabetic women were excluded. This review includes references published between January 2000 and July 2012. 

## 2. Results 

Using the above mentioned keywords for searches of the PubMed database, 256 studies were detected. After applying the inclusion/exclusion criteria, 16 studies remained. An additional 3 studies were identified from the literature obtained. Among the intervention studies that combined physical activity, nutrition and recommendations regarding healthy GWG (Table 2), 9/13 (69%) were successful at decreasing mean GWG and/or preventing excessive GWG based on the 1990 (Table 2(a)) or 2009 (Table 2(b)) IOM guidelines [44, 55–62] and 4/13 (31%) were unsuccessful [63–66]. Among intervention studies that included a physical activity component alone (Table 3), 2/6 (33%) were only partially successful at reducing mean GWG and/or preventing excessive GWG based on the IOM 1990 (Table 3(a)) or 2009 (Table 3(b)) guidelines [67, 68] and 4/6 (67%) were unsuccessful [69–72].

### 2.1. Effect of Prenatal Lifestyle Interventions on Gestational Weight Gain

#### 2.1.1. Successful Interventions at Reducing Mean Gestational Weight Gain

Asbee at al. [55] (Table 2(a)) examined the effect of a prenatal intervention on GWG in women of various pre-pregnancy BMI categories (normal weight, overweight, and obese women). This randomised controlled trial (RCT) included detailed recommendations regarding dietary intake, physical activity, and appropriate GWG based on the women's pre-pregnancy BMI. The authors found lower GWG in the intervention group (16.2 ± 7.0 kg) compared to the control group (13.0 ± 5.7, *P* = 0.01), regardless of BMI status. Although the participants received specific recommendations about dietary intake and physical activity, compliance to these recommendations was not reported. In addition, no information was given regarding physical activity levels and eating habits of the control group. Two other successful interventions included only women who were overweight and/or obese prior to pregnancy. Shirazian et al. [57] (Table 2(b)) conducted a prenatal intervention that included six seminars and one-on-one counseling sessions (*n* ≥ 5) aimed at promoting healthy eating and encouraging walking (food diaries and pedometers were provided to the participants) and educating the women on obesity during pregnancy. The intervention group gained less weight than the control group (8.1 ± 7.4 kg versus 15.4 ± 7.5 kg, resp.; *P* = 0.003). Unfortunately, eating and physical activity habits of the women during the intervention were not reported, although the participants received tools (diaries and pedometers) to monitor their daily dietary intake and physical activity levels. Further, physical activity levels and eating habits of the control group were not reported. Finally, the study of Nascimento et al. [68] (Table 3(b)) included weekly supervised aerobic dance classes of moderate-intensity, combined with recommendations about weekly physical activity level and healthy GWG. The authors reported lower mean GWG in the intervention group (10.0 ± 1.7 kg) compared with the control group (16.4 ± 3.9 kg, *P* = 0.001) but only in overweight women (17.5% of the sample) [68]. Sixty-three percent of the women were compliant with the exercise intervention and accumulated a mean of 80 ± 49 minutes of walking every week and 57 ± 22 minutes of exercise from the study protocol. However, no information was provided regarding physical activity levels in overweight and obese women separately. Moreover, the authors did not report physical activity levels for the control group. 

Taken together, although these 3 studies were successful at reducing mean GWG, none of them produced statistically significant differences in adherence to IOM recommendations, based on the 1990 [55] or 2009 [57, 68] guidelines.

#### 2.1.2. Successful Interventions at Preventing Excessive Gestational Weight Gain Based on the IOM Guidelines

**Studies Using the 1990 IOM Guidelines**

Polley et al. [58] (Table 2(a)) conducted a behavioral intervention with stepped care (increasing one-on-one care if needed) in low-income women of various pre-pregnancy BMI categories (normal weight, overweight, and obese women). The intervention group received education about weight gain, healthy eating and exercise, and individual graphs of their weight gain. The intervention was successful only among normal weight women (55% of the sample), with more women not exceeding the IOM guidelines in the intervention versus control group (67% versus 42%, resp.; *P* < 0.05) [58]. Changes in dietary intake and exercise expenditure were assessed by questionnaires pre- and post-intervention and showed that normal weight women in the intervention group decreased their fat intake whereas no changes were found in normal weight women in the control group. Regarding physical activity levels, no changes were found in any of the groups [58]. A larger study, based on a clinical component and a by-mail education program targeting healthy eating, physical activity and GWG, with an emphasis on self-monitoring, was successful but only in women of low income (41% of the sample) [61] (Table 2(a)). Among women of normal weight, 71% of those who participated in the intervention did not exceed the IOM guidelines compared to only 55% (*P* = 0.05) of the historical cohort. Among the overweight women, 56% of those in the intervention group did not gain excessively compared to only 28% (*P* = 0.04) of the historical cohort. Mean GWG, as well as nutrition and physical activity habits of these low-income women were not reported. Finally, Phelan et al. [59] (Table 2(a)) examined the effect of a low-intensity, partially mail-based behavioral intervention targeting dietary intake, physical activity, and weight gain monitoring. The authors found that their intervention was successful but only in women of normal weight prior to pregnancy, with 60% of the women who participated in the intervention not gaining excessively compared with only 48% of those receiving standard prenatal care (*P* = 0.003). Although the participants received food records and pedometers, nutrition and physical activity habits of the women during the intervention were not reported. Similarly, information regarding lifestyle habits of the control group was not reported.

**Studies Using the 2009 IOM Guidelines**

Hui et al. [60] (Table 2(b)) conducted an intervention that combined personalized dietary counseling, weekly supervised exercise sessions and home-based exercises (VHS and DVD). Women of different pre-pregnancy BMI categories were included in the study. Results showed that 65% of the women who followed the intervention did not gain above the 2009 IOM guidelines compared with only 45% of those in the control group (*P* = 0.008). Only 56% of the women completed a 3-day food intake record at baseline and 2 months after enrolment and the results showed lower energy and fat intake in the intervention group compared to the control group in response to the intervention. Further, 94% of the women completed physical activity questionnaires and the results showed that women in the intervention group increased their physical activity levels and were active more than twice/week for more than 20 minutes/session 2 months after enrolment. No changes in physical activity levels were found in the control group. All women in the intervention group self-reported home exercise for 3–5 times/week during the study.

Taken together, although these 4 studies were successful at preventing excessive GWG based on the 1990 [58, 59, 61] or 2009 [60] IOM guidelines, they did not show a significant reduction in mean GWG.

#### 2.1.3. Successful Interventions at Reducing Mean Gestational Weight Gain and Preventing Excessive Gestational Weight Gain Based on the IOM Guidelines

Only 4/19 (21%) intervention studies were successful at both reducing mean GWG and preventing excessive GWG based on the IOM guidelines [44, 56, 62, 67].

**Studies Using the 1990 IOM Guidelines**

Mottola et al. [44] (Table 2(a)) used a Nutrition and Exercise Lifestyle Intervention Program (NELIP) for overweight and obese pregnant women. This single-arm intervention included a personalized meal plan combined with a supervised walking program of mild-intensity 3 to 4 times/week. This intervention was highly successful, with 80% of the women not exceeding recommended GWG while on NELIP. Mean GWG during the intervention was 6.8 ± 4.1 kg (0.38 ± 0.2 kg/week) [44]. During the intervention, the women walked 2.84 ± 0.87 times per week and dietary intake assessments showed a significant decrease in daily intake of total energy (from 2,228.0 ± 474.6 to 1,900.2 ± 343 kcal) and carbohydrate (from 318.5 ± 155.1 to 259.1 ± 93.9 g) and an increase in the percent of daily energy from protein (16.9% ± 2.4% to 18.4% ± 2.3%).

**Studies Using the 2009 IOM Guidelines**

Claesson et al. [56] (Table 3(b)) examined the effect of an intervention that included only women who were obese prior to pregnancy. The cornerstone in the intervention was a motivational interview/talk in early pregnancy, focusing on behavior change. During the intervention, the women were invited to a 30-minute individual session every week for motivational talks and weight gain control. Further, the women were invited to attend aqua aerobic classes 1-2 times/week. Women in the intervention group gained less weight than those in the control group (8.7 ± 5.5 kg versus 11.3 ± 5.8, resp., *P* < 0.001) and were more likely to gain less than 7.0 kg (study goal) (36% versus 21%, resp., *P* = 0.003) [56]. Behavior changes in response to the intervention were not reported, nor attendance for the aqua aerobic classes. Similarly, no information was given regarding the lifestyle habits of the control group. The intervention of Vinter et al. [62] (Table 2(b)) included personalized dietary counseling provided on 4 occasions during the study, supervised exercise sessions offered once per week, with free access to a fitness center for the duration of the study, and 4–6 group sessions given to improve participants' integration of physical activities in pregnancy and daily life. Mean weight gain was 7.0 kg (4.7–10.6 kg) in the intervention group and 8.6 kg (5.7–11.5 kg) in the control group (*P* = 0.01) [62]. Although more women in the intervention group versus control group did not exceed the 2009 IOM recommendations, the difference was statistically not significant (65% versus 53%, resp., *P* = 0.058). Compliance with the nutrition counseling sessions was very good, with 92% of the women having completed all 4 nutrition counseling sessions. Eighty-five percent of the women in the intervention group and 21% of those in the control group reported that their participation in the study resulted in more healthy eating habits. However, no information regarding changes in dietary intake of the women was provided by the authors. Attendance for physical activity was more difficult to maintain, with only 56% of the women having attended the aerobic classes for at least half of the lessons. Seventy-eight percent of the women in the intervention group versus 65% of those in the control group engaged in leisure time physical activities during pregnancy. However, the frequency, duration, and intensity of these activities were not reported. Finally, Haakstad et Bø [67] (Table 2(a)) investigated the effect of a 12-week exercise program in women of different pre-pregnancy BMI categories (75% were of normal weight). The program consisted of 60 minutes of supervised aerobic dance performed at least twice/week. The women were asked to exercise 30 minutes/day on the other days of the week. The intervention was found to be effective only in women who attended 2 dance classes per week (27% of the exercising group), with a mean weight gain of 11.0 ± 2.0 kg in the exercising women and 13.0 ± 4.0 kg in the control women (*P* = 0.01). All exercising women gained within the recommendations compared to 62% of the control women (*P* = 0.006).

#### 2.1.4. Unsuccessful Interventions at Reducing Mean Gestational Weight Gain and Preventing Excessive Gestational Weight Gain Based on the IOM Guidelines

A large number of intervention studies were unfortunately not successful at reducing mean GWG and preventing excessive GWG based on the IOM guidelines.

**Studies Using the 1990 IOM Guidelines**

Gray-Donald et al. [64] (Table 2(a)) developed a community-based intervention for the Aboriginal Cree population, based on the social learning theory. Components of this intervention were pamphlets about nutritional choices, supermarket tours and cooking demonstrations, exercise and walking group sessions, and individual counseling. The intervention, that included women of different pre-pregnancy BMI categories (mean BMI of 30.0 ± 6.5 kg/m^2^), had no effect on total maternal weight gain and did not prevent excessive GWG based on IOM guidelines. Eating and physical activity habits did not improve. In fact, self-reported sedentary behaviors were significantly higher in the intervention group (61%) compared with the control group (23%) [64]. Guelinckx et al. [65] (Table 2(a)) examined the effect of an intervention study that was based on a brochure (passive intervention) or on active education (active intervention) about GWG, dietary habits and physical activity in women who were obese prior to pregnancy. Although improvements in dietary habits were observed in both intervention groups, both interventions were unsuccessful at reducing mean GWG and preventing excessive GWG. Physical activity levels were similar in all three groups and decreased significantly throughout pregnancy. Finally, Kinnunen et al. [63] (Table 2(a)) examined the effect of an intervention that included 4 individual counseling sessions on healthy diet and 5 counseling sessions to help increasing leisure-time physical activity (LTPA), and designing an individual activity plan to achieve a healthy GWG. In addition, the participants had the opportunity to join group exercise sessions once a week for 45–60 minutes. The women, who were of various pre-pregnancy BMI categories (75% were of normal weight) exhibited similar GWG (intervention group, 14.6 ± 5.4 kg versus control group, 14.3 ± 4.1 kg, resp.). Surprisingly, the authors reported a trend in a higher number of women not exceeding the IOM guidelines in the control group compared to the intervention group (70% versus 54%, *P* = 0.053). However, the intervention improved dietary habits (increase in the intake of vegetables, fruits, and berries) but had no effect on LTPA levels.

**Studies Using the 2009 IOM Guidelines**

Lindholm et al. [66] (Table 2(b)) conducted a single-arm intervention for obese women who were invited to an initial individual consultation with a dietician and then to meet with a midwife once every two weeks and to attend 2 group-support sessions. In addition, the intervention included water aerobic classes once per week and the women were asked to exercise 30 minutes/day on other weekdays. Excessive GWG (i.e., >6.0 kg) was prevented in only 56% of the women, with a mean GWG of 6.9 ± 6.4 kg. No information was given regarding improvements in eating and physical activity habits of the women in response to the intervention. Further, attendance to the water aerobic classes was not reported. Finally, 3 studies that included only an exercise intervention were found to be unsuccessful. Barakat et al. [69, 72] (Table 3(b)) published two studies examining the effect of a supervised exercise program performed 3 times/week for 35–40 minutes/session, one focusing on light resistance and toning exercises and the other on aerobic exercises (1/week) and aquatic activities (2/week). Both interventions included women of different pre-pregnancy BMI categories (mean BMI in the normal range). Although adherence to the exercise program was >85%, similar GWG was found in the intervention and control groups. No information was given regarding whether the intervention was successful at preventing excessive GWG based on the 2009 IOM guidelines. Similarly, a supervised water aerobic exercise program of moderate intensity performed 3 times/week for 50 minutes/session had no effect on GWG in sedentary pregnant women of different pre-pregnancy BMI categories [70] (Table 3). The women participated in a mean of 25 exercise sessions during this 15-week intervention and GWG was similar in the intervention and control groups (14.3 ± 2.1 kg and 15.1 ± 1.6 kg, resp.). Adherence to IOM guidelines was not reported by the authors. Finally, Yeo [71] (Table 3(a)) compared the effect of a supervised walking program to a stretching program on preeclampsia risk factors, including excessive GWG, in women of different BMI categories prior to pregnancy (81% were obese). The intervention consisted of 40 minutes of stretching or moderate-intensity walking, 5 times/week. Mean GWG was similar in both groups (15.4 ± 5.9 kg in the women who walked and 15.9 ± 6.8 kg in the stretching women), although the walkers tracked more daily steps compared to the stretching group, with an average of 7, 790 ± 3, 890 and 5, 355 ± 3, 044 steps per day, respectively (*P* = 0.0002). Even if the step difference between the groups was statistically significant, a difference of approximately 2,500 steps per day may not have been enough to impact on GWG. Noteworthy, 2,500 steps is equivalent to approximately 25 min of moderate-intensity walking, implying that the women were not compliant with the exercise program recommending 40 min of moderate-intensity walking.

#### 2.1.5. Summary and Points to Discuss

Based on the studies presented above, it appears that prenatal lifestyle interventions promoting healthy eating and physical activity habits were more successful at decreasing mean GWG and/or preventing excessive GWG compared to those including an exercise component alone (69% versus 33%). This suggests that the same principle may apply to the prevention of excessive GWG as in weight loss: the major component is in dietary changes and the role of physical activity is to support and maintain these achievements [73]. 

However, there are still a large number of prenatal lifestyle interventions that were unsuccessful and it is important to discuss the factors that may explain their ineffectiveness in order to design future successful prenatal lifestyle interventions. These factors may be numerous and include characteristics of the women (parity, socioeconomic status, maternal pre-pregnancy BMI, pre-pregnancy physical fitness levels, pre-pregnancy lifestyle habits), the design of the intervention, the gestational age at which the intervention was initiated, the components of the intervention (nutrition, physical activity, behavioral change), the frequency and intensity of physical activity, the type of intervention (i.e., phone-based, mail-based, or supervised intervention), the frequency of the interaction with the women, the contexts or providers of counseling, and, of course, compliance with the intervention. 

There is good evidence to suggest that pre-pregnancy BMI is an important factor influencing the success of an intervention and that it should be taken into consideration when designing a prenatal lifestyle intervention. For example, overweight and obese pregnant women likely had unhealthy eating habits and a sedentary lifestyle before becoming pregnant. Asking them to make behavioral changes while participating in a prenatal lifestyle intervention may be challenging. Moreover, they may have body weight issues and low self-esteem. Because of these factors, overweight and obese pregnant women may need more support and encouragement during an intervention to be able to overcome the barriers they may face in order to improve their lifestyle habits and be successful in limiting their weight gain. Accordingly, intervention studies that included overweight and obese women and were successful at reducing mean GWG and preventing excessive GWG were based on frequent individualized nutrition counseling sessions and discussions regarding healthy weight gain throughout pregnancy, combined with supervised exercise sessions [44, 56, 62]. On the other hand, intervention studies based only on written or oral recommendations regarding healthy lifestyle habits during pregnancy and of less frequent interactions were unsuccessful at reducing mean GWG or preventing excessive GWG in overweight and obese women [59, 65]. The way of approaching behavioral change is also of paramount importance, especially in overweight and obese women. Only few prenatal lifestyle interventions included any theoretical background as a basis for behavioral change. However, we may speculate that interventions based on frequent interactions with the participants and discussions regarding healthy weight gain included behavioral change objectives, which may have contributed to the success of these interventions. The background of the providers of counseling may also have influenced the success of the intervention. Counseling provided by the same team of healthcare professionals throughout the intervention (nurse, midwife, dietician, kinesiologist, or psychologist) may have had a stronger impact on helping the women to improve their lifestyle habits and be successful in limiting their weight gain, emphasizing a team approach. 

Another factor that may have contributed to the mixed results of prenatal lifestyle interventions may be the use of the 1990 versus 2009 IOM GWG guidelines, although we cannot draw conclusions as to whether studies using the 1990 or 2009 IOM guidelines were more likely to be successful at preventing excessive GWG. However, with the increased prevalence of maternal obesity and the new GWG guidelines that are more restrictive, especially for obese women, we may expected that upcoming studies will have to provide more interactive and frequent counseling sessions to be successful at preventing excessive GWG in obese women.

Finally, an important methodological issue that was identified in several prenatal lifestyle interventions presented above is that although they all included a physical activity component, combined or not with nutritional advice, they did not assess dietary intake and physical activity levels of the participants pre- and post-intervention. Similarly, some interventions provided food diaries and pedometers to the participants in order to encourage them to monitor their daily dietary intake and physical activity levels but the data collected using these tools were not reported by the authors. This is important missing information because researchers need to identify from previous successful interventions what contributed to their success in order to design future successful prenatal lifestyle interventions. Consequently, it is highly important that researchers document the important/effective aspects of their intervention by examining whether the participants were compliant with the recommendations given during the intervention and whether they had modified eating and/or physical activity habits. Further, it is important to examine eating and/or physical activity habits of the control group. The few authors who reported data regarding the impact of their intervention on changes in nutrition and physical activity habits of the participants have found that decreasing fat intake [58, 60], decreasing carbohydrate intake while increasing protein consumption [44], and increasing physical activity levels to meet physical activity recommendations during pregnancy [44, 60, 67, 68] helped to achieve a healthy GWG. 

However, compliance is always difficult to interpret and the authors should report as much information regarding compliance as possible. For example, if compliance with physical activity recommendations is defined as being active 3 times per week, the authors should not only report the mean number of weekly exercise sessions performed by the women but also the percentage of women having been active 3 times per week. In the case where there is few but exceptionally well-adhered women who were active 6 times per week, these women will pull the group average up. The reader will then consider that the intervention was effective because all women were compliant with physical activity recommendations. In fact, the few but exceptionally well-adhered women were mostly responsible for the positive results. However, compliance with the physical activity component of a prenatal lifestyle intervention was found to be a major problem. Factors such as concerns for the safety of the baby, physical limitations, and lack of energy, motivation, or resources may contribute to the low compliance with the physical activity program/recommendations. The inclusion of supervised exercise sessions may help to favor compliance with the physical activity component of the intervention and achievement of healthy weight gain. It would allow frequent interactions with the women during which physical activity-related behavior change objectives may be provided and emphasis put on the safety and health benefit of physical activity during pregnancy. 

Taken together, differences in the characteristics of the women, the nutritional, physical activity, and behavioral change approaches used make the comparison of different interventions difficult and the identification of the most effective ways to prevent GWG challenging. Further research is needed to identify the effective aspects of prenatal lifestyle interventions, especially for overweight and obese women, in order to support the current evidence suggesting that prenatal lifestyle interventions may play an important role in the prevention of excessive GWG.

### 2.2. Does Limiting Gestational Weight Gain Decrease the Incidence of High Infant Birth Weight?

Although the prevention of excessive GWG is important to decrease the incidence of high infant birth weight, 4/19 (21%) of the intervention studies did not report infant birth weight. Among the intervention studies that were successful in decreasing mean GWG or preventing excessive GWG based on the IOM recommendations, two studies did not report infant measurements at birth [55, 67] and seven reported no differences in infant birth weight or in the incidence of low (<2,500 g) or high (>4,000 g) birth weight between the intervention and control groups [56–61, 68]. Only two studies showed a significant effect of limiting GWG on infant weight at birth. Mottola et al. [44] (Table 2(a)) reported that in the overweight women, a lower percentage of babies born weighing between 4,000 g and 4,500 g was found in the intervention group compared to the historical control cohort (3.2% versus 18%, resp.; *P* = 0.048). However, similar overall mean birth weight was found between the intervention group (3, 590 ± 500 g) and the historical control cohort (3, 560 ± 600 g; *P* > 0.05). Also, no babies born to the NELIP women weighed less than 2,500 g, whereas 3.5% of the babies born to the historical control cohort were in this weight category. Conversely, Vinter et al. [62] (Table 2(b)) surprisingly found higher birth weights in the intervention group (3,742 g) compared to the control group (3,593 g; *P* = 0.039), with a trend in higher frequency of high birth weight babies (>4,000 g) in the intervention group compared to the control group (32% versus 25%; *P* = 0.07). 

#### 2.2.1. Summary and Points to Discuss

Based on these prenatal lifestyle intervention studies, conclusions suggesting that decreasing GWG or preventing excessive GWG may be linked to lower incidence of high birth weight cannot be drawn, with the exception of one study [44]. Noteworthy, the main outcome of the prenatal lifestyle intervention studies presented above was preventing excessive GWG, with secondary outcomes being preventing high infant birth weight and pregnancy complications. Thus, it is likely that these studies were underpowered to find a significant effect on these outcomes. Future larger and well-controlled intervention studies are therefore warranted to examine the effect of the promotion of healthy lifestyle habits and adequate GWG on newborn weight. 

Moreover, future studies should assess body composition of the neonates. First, even if infant weight at birth is similar between the intervention and control groups, differences in body fat distribution might exist and have a long-term impact on the metabolic health of the infant. Second, if a higher birth weight is observed, it is highly important to evaluate whether this is due to higher lean mass or fat mass. Vinter et al. [62], who found a higher infant weight at birth in the intervention group compared to the control group, mentioned the absence of information regarding body composition of the neonates as a limitation of their study. They also suggested that their findings may be explained by the stimulation of placenta development in response to maternal exercise, which has been previously reported [74]. Another explanation may be the time-specific effect of exercise on fetal growth, as discussed in a recent review paper [75]. Beginning an exercise program in early pregnancy (i.e., first trimester) has been reported to have a stimulatory effect on placenta growth and function, which may increase infant birth weight [75]. Although this may be a beneficial adaptation in lean and physically active women, it may have a less desirable effect of promoting excess fetal growth in overweight/obese women. Interestingly, the intervention of Vinter et al. [62] started early in pregnancy (10–14 weeks) and the data support this time-specific effect of exercise on fetal growth. Initiating an exercise program for overweight/obese women during the second trimester of pregnancy may be more beneficial in preventing excess fetal growth, as shown by the study of Mottola et al. [44].

The timing of excessive GWG during pregnancy also deserves more attention. Indeed, excessive GWG that occurs early in pregnancy may not have the same influence on fetal growth and infant weight at birth compared to excessive GWG occurring later in pregnancy. For example, excessive GWG, especially during the first trimester of pregnancy, has been found to be associated with an increased risk of gestational diabetes [76, 77], suggesting that timing of excessive GWG may be related to different patterns of metabolic change affecting glucose metabolism regulation as well as fetal growth. 

Finally, it is important to consider that even if prenatal lifestyle interventions were successful at limiting GWG or preventing excessive GWG but had no effect on infant outcome at birth, there still may be a beneficial effect on the long-term health of the infant. As suggested by the fetal programming (Barker's) hypothesis [78], the prenatal period is a unique physiological window in which maternal and fetal adaptations can have major consequences for the long-term health and well-being of the offspring. More research is needed to examine the effect of prenatal lifestyle interventions on the infant weight trajectory during childhood and adulthood. Moreover, although epidemiological studies have suggested that excessive GWG is associated with an increased risk for the infant of becoming overweight/obese later in life [8, 13, 16, 17], the molecular mechanisms through which the exposure to excessive GWG translates into the development of obesity among offspring is currently unknown. Epigenetics (i.e., changes in gene expression) has been suggested as a very likely mechanism. Genes and metabolic pathways showing epigenetic dysregulation in response to the exposure to excessive GWG are likely involved in obesity development among children. The identification of these genes and metabolic pathways is of high interest and may have the potential in the long term to foster the development of improved prevention programs and treatments.

### 2.3. Does Limiting of Excessive Gestational Weight Gain Prevent Postpartum Weight Retention?

Finally, one of the objectives of preventing excessive GWG is to optimize maternal outcomes, such as preventing maternal weight retention. However, only 7/19 (37%) of the prenatal lifestyle intervention studies followed the women after delivery [44, 56, 58, 59, 61, 64, 67]. Gray-Donald [64] and Polley et al. [58] (Table 2(a))** **found similar weight retention at the first postnatal visit in women who followed the intervention and in those who were in the control group. Haakstad and Bø [67] (Table 3(b))**  **reported lower weight retention at 8–12 weeks postpartum in women who were compliant with the prenatal exercise program compared to those in the control group (0.8 ± 1.7 kg versus 3.3 ± 4.1 kg, resp.; *P* = 0.001). Similarly, Claesson et al. [56] (Table 2(b))**  **found a mean weight retention of −2.15 ± 5.88 kg and 0.75 ± 5.34 kg at 10–12 weeks postpartum in obese women who followed the intervention and in those who received standard prenatal care, respectively (*P* < 0.001). In addition, Mottola et al. [44] (Table 2(a))**  **showed that overweight/obese women who participated in the NELIP retained a mean of 2.2 ± 5.6 kg at 2 months postpartum, with 53% of the women being within 2.0 kg of pre-pregnancy body mass. Phelan et al. [59] (Table 2(a)) reported that normal weight and overweight/obese women prior to pregnancy who received the intervention were more likely to be at, or below, their preconception weight at 6 months postpartum than women who received standard prenatal care (OR: 2.1; 95% CI: 1.3, 3.5; *P* = 0.005). Finally, Olson et al. [61] found that a significantly smaller proportion of intervention group women retained 2.27 kg or more at 1-year postpartum in the low-income and overweight BMI group (25% versus 55%; *P* = 0.04). 

#### 2.3.1. Summary and Points to Discuss

Taken together, these findings support the importance of promoting a healthy lifestyle during pregnancy to reduce postpartum weight retention. However, because only 32% of prenatal lifestyle intervention studies aiming at preventing excessive GWG followed the women into the postpartum period, future studies should include follow-up in their design. Further, studies are also needed to examine whether the promotion of healthy lifestyle habits during pregnancy has a long-lasting effect in both mother and offspring, beyond the postpartum period. 

## 3. Conclusion

Obesity is reaching epidemic proportions in today's society and women of childbearing age are at an increased risk for developing this disease because of excessive weight gain during pregnancy and weight retention after birth. Further, offspring of women who gained excessively during pregnancy are at an increased risk of becoming overweight and obese in the long term. Promoting a healthy lifestyle during pregnancy and providing recommendations regarding healthy weight gain, especially in overweight and obese women, become therefore increasingly important in the context of the prevention of obesity. Several prenatal lifestyle interventions have been published but due to several issues discussed in this review, it is difficult to make any conclusions about the most effective way to prevent excessive GWG, reduce the incidence of small or large babies and prevent weight retention. However, prenatal interventions combining a nutritional counseling, supervised physical activity sessions, and a behavioral change approach might be the most successful (Table 4). Noteworthy, this review is restricted to studies published in English, which included mainly Caucasian women. It is possible that the most effective way to prevent excessive GWG is different among women of other ethnicities. 

Although the role of prenatal physical activity to prevent excessive GWG has been recently questioned, the prevailing literature indicates that women who were compliant with the physical activity program and met the physical activity recommendations were more likely to achieve appropriate GWG and return to their pre-pregnancy body mass after delivery. Consequently, the role of physical activity during pregnancy per se is not the issue. Instead, it is the designs of the physical activity approaches that may not have been adequate for preventing excessive GWG and/or protocols that were not structured to achieve desired compliance. Only few interventions included any theoretical background as a basis for behavioral change and such approach may help to increase compliance with physical activity program/recommendations. Rigorous scientific research, designed to increase compliance of the women, is still required to make an informed decision about the role of physical activity in the prevention of excessive GWG. In addition, research is needed to examine the long-lasting effect of the prevention of excessive GWG on the risk of obesity for the mother and offspring. Finally, investigating the molecular mechanisms through which the exposure to excessive GWG translates into the development of obesity among offspring (i.e., epigenetic factors) may help to better understand the aetiology of obesity and to develop more efficient prevention intervention and treatment.

## Figures and Tables

**Table 1:** Gestational weight gain recommendations based on the 1990 and 2009 Institute of Medicine guidelines [5, 79].

IOM 1990		IOM 2009		
Pre-pregnancy BMI category	Recommended^b^ range of total weight gain	Pre-pregnancy BMI category	Mean^a^ rate of weight gain in the 2nd and 3rd trimester	Recommended^b^ range of total weight gain
BMI < 19.8 kg/m^2^ (low)	12.5–18.0 kg	BMI < 18.5 kg/m^2^ underweight	0.5 kg/week	12.5–18.0 kg
BMI 19.8–26.0 kg/m^2^ (normal)	11.5–16.0 kg	BMI 18.5–24.9 kg/m^2^ normal weight	0.4 kg/week	11.5–16 kg
BMI 26.1–29.0 kg/m^2^ (high)	7.0–11.5 kg	BMI 25.0–29.9 kg/m^2^ overweight	0.3 kg/week	7.0–11.5 kg
BMI > 29.0 kg/m^2^ (obese)	at least 6.0 kg	BMI ≥ 30^c^ kg/m^2^ obese	0.2 kg/week	5.0–9.0 kg

^a^Rounded values.

^b^Calculations assume a total of 0.5–2.0 kg weight gain in the first trimester.

^c^A narrower range of weight gain may be advised for women with a pre-pregnancy BMI of 35.0 kg/m^2^ or greater. Individualized advice is recommended for these women.

**Table:** (a) Prenatal intervention studies using the 1990 IOM gestational weight gain recommendations

Reference, sample characteristics	Treatment		Gestational weight gain (GWG) (kg)			Maternal weight retention postpartum (PP) (kg)			Infant birth weight (g)		
	Control	Intervention	Control	Intervention	*P* value	Control	Intervention	*P* value	Control	Intervention	*P* value
Gray-Donald et al. [64] Design: Time-series control trial *N* = 219 *C* = 107 *I* = 112 *Country* Canada *Ethnicity* Cree women *Age* *C* = 23.8 ± 5.86 *I* = 24.3 ± 6.29 *SES* NA *Prepreg BMI* *C* = 29.6 ± 6.45 *I* = 30.8 ± 6.85 *Parity* *C* = 73% nulliparous *I* = 31% nulliparous *Gestational age at recruitment* *C* = 18.5 ± 6.9 *I* = 17.1 ± 7.1 *Duration of study* until delivery	Standard prenatal care	Community-based intervention for specific population (Aboriginal Cree) Based on the social learning theory *Nutrition* Pamphlets about nutritional choices, supermarket tours and cooking demonstrations, individual counseling *Physical activity* Exercise/walking groups *Other* Advice to stay within GWG recommendations Delivered by dieticians and health workers	*At delivery* 13.2 ± 8.3 *Weekly GWG* BMI ≤ 29: 0.63 ± 0.32 BMI > 29: 0.44 ± 0.30 *Adherence to 1990 IOM* NA	*At delivery* 12.0 ± 6.4 *Weekly GWG* BMI ≤ 29: 0.62 ± 0.27 BMI > 29: 0.44 ± 0.24 *Adherence to 1990 IOM* NA	0.29 NS NS	*At 6 weeks PP* 7.4 ± 8.5	*At 6 weeks PP* 6.1 ± 6.7	>0.05	3,741 ± 523 <2,500 g *n* = 2 (2%) >4,000 g *n* = 31 (30%)	3,686 ± 686 <2,500 g *n* = 3 (3%) >4,000 g *n* = 37 (35%)	NS NS NS

Polley et al. [58] Design: RCT *N* = 110 *C* = 53 *I* = 57 *Country* United States *Ethnicity* 39% black 61% white, *Age* 25.5 ± 4.8 *Parity* 47% nulliparous *SES* low income *Gestational* *age at recruitment* 14.5 ± 3.1 *Duration of study* NA	Standard prenatal care	*Nutrition* Bi-weekly newsletters on healthy eating *Physical activity* Bi-weekly newsletters on healthy exercise habits, increasing walking and developing a more active lifestyle *Other* Written and oral info about appropriate GWG, personalized graphs showing GWG Delivered by trained staff	*At 36–40 weeks* 13.8 ± 5.4	*At 36–40 weeks* 14.5 ± 7.1	NS	NA	NA	—	NA	NA	—

Normal weight group *C* = 31 *I* = 30 *Prepreg BMI* *C* = 22.5 ± 2.0 *I* = 22.8 ± 1.9			*At 36–40 weeks* 16.4 ± 4.8 *Adherence to 1990 IOM* 42% did not exceed	*At 36–40 weeks* 15.4 ± 7.1 *Adherence to 1990 IOM* 67% did not exceed	NS **<0.05**	*At 8 ± 7 w PP* 6.2 ± 4.5	*At 8 ± 7 w PP* 4.4 ± 5.4	NS	3,226 <2,500 g *n* = 3 (10%) >4,000 g *n* = 0	3,133 <2,500 g *n* = 4 (13%) >4,000 g *n* = 1 (3%)	NS NS NS

Overweight group *C* = 22 *I* = 27 *Prepreg BMI* *C* = 34.1 ± 7.2 *I* = 31.4 ± 6.0			*At 36–40 weeks* 10.1 ± 6.2 *Adherence to 1990 IOM * 68% did not exceed	*At 36–40 weeks* 13.6 ± 7.2 *Adherence to 1990 IOM* 41% did not exceed	NS 0.09	*At 8 ± 7 w PP* 0.3 ± 7.0	*At 8 ± 7 w PP* 3.6 ± 5.6	NS	3,349 <2,500 g *n* = 2 (9%) >4, 000 g *n* = 0	3,283 <2,500 g *n* = 1 (4%) >4,000 g *n* = 0	NS NS NS

Olson et al. [61] Design Historical cohort *N* = 560 *C* = 381 *I* = 179 *Country* United States *Ethnicity* 96% white *Age:* *C* = 94% 20–40 yr *I* = 94% 20–40 yr *Parity* *C* = 41% nulliparous *I* = 41% nulliparous *SES* *C* = 43% low income *I* = 37% low income *Prepreg BMI* 75% normal weight in early pregnancy *Gestational age at recruitment* <27 weeks *Duration of study* NA	Standard prenatal care	*Nutrition* Tips and newsletters about healthy eating, “health checkbook” to help self-monitoring diet *Physical activity* Tips and newsletters about PA during pregnancy *Other* Guidance about self-monitoring of GWG, newsletters about GWG Delivered by healthcare providers	*At delivery* 14.8 ± 4.7 *Adherence to 1990 IOM* 55% did not exceed **Sub-group,** **Low SES** *Adherence to 1990 IOM* NW 55% OW 28% did not exceed	*At delivery* 14.1 ± 4.5 *Adherence to 1990 IOM* 59% did not exceed **Sub-group,** **Low SES** *Adherence to 1990 IOM* NW 71% OW 56% did not exceed	0.09 0.30 **0.05** **0.04**	*At 1 year* 1.3 ± 5.6 **Sub-group, Low SES** *Weight retention ≥2.27 kg* NW 40% OW 55%	*At 1 year* 0.6 ± 4.8 **Sub-group, Low SES** *Weight retention ≥2.27 kg* NW 37% OW 25%	0.14 >0.05 0.04	3,621 (2,060–5,486) <2,500 g *n* = 3 (1%) >4,000 g *n* = 83 (22%)	3,610 (2,475–5,345) <2,500 g *n* = 2 (1%) >4,000 g *n* = 32 (18%)	NS NS NS

Kinnunen et al. [63] Design non RCT *N* = 105 *C* = 56 *I*= 49 *Country* Finland *Ethnicity* Finish *Age* *C* = 28.8 ± 4.1 *I* = 27.6 ± 4.5 *Parity* 100% nulliparous *SES* *C* = 36% Basic/secondary education *I* = 57% Basic/secondary education *Prepreg BMI* *C* = 22.3 ± 2.1 *I* = 23.7 ± 3.9 *Gestational age at recruitment* 8-9 weeks *Duration of study* until 37 weeks of gestation	Standard prenatal care	*Nutrition* 4 individual counseling sessions on healthy diet *Physical activity* 5 counseling sessions to increase LTPA and design an individual activity plan. Possibility to join group exercise sessions once a week for 45–60 min *Other* Information on GWG recommendations Delivered by nurses	*At 36-37 weeks* 14.3 ± 4.1 *Adherence to 1990 IOM* 70% did not exceed	*At 36-37 weeks* 14.6 ± 5.4 *Adherence to 1990 IOM* 54% did not exceed	0.77 **0.053**	NA	NA	—	≥4,000 g *n* = 8 (15%)	≥4,000 g *n* = 0	**0.006**

Asbee et al. [55] Design RCT *N* = 100 *C* = 43 *I* = 57 *Country* United States *Ethnicity* mixed *C* = 55% Hispanic *I* = 58% Hispanic *Age* *C* = 26.4 ± 5.0 *I* = 26.7 ± 6.0 *Parity* *C* = 44% nulliparous *I* = 46% nulliparous *SES* *C* = 65% high school or < *I* = 68% high school or < *Prepreg BMI* *C* = 25.6 ± 5.1 *I* = 25.5 ± 6.0 *Gestational age at recruitment* *C* = 13.6 ± 3.6 *I* = 13.7 ± 3.6 *Duration of study* until delivery	Standard prenatal care	*Nutrition* Nutrition plan containing 40% carbohydrate 30% protein 30% lipid *Physical activity* Recommendations *Frequency *3–5/week *Intensity * moderate-intensity exercise *Other* Information about GWG guidelines Delivered by dieticians	*At delivery* 16.2 ± 7.0 *Adherence to 1990 IOM* 49% did not exceed	*At delivery* 13.0 ± 5.7 *Adherence to 1990 IOM* 61% did not exceed	**0.01** 0.21	NA	NA	—	NA	NA	—

Mottola et al. [44] Design Case (historical cohort)-control matched design *N* = 325 *C* = 260 *I* = 65 *Country* Canada *Ethnicity* >50% aboriginal *Age:* *C* = 31.9 ± 3.5 *I* = 32.4 ± 4.0 *Parity* *C* = 38% nulliparous *I* = 38% nulliparous *SES* na *Prepreg BMI* *C* = 33.4 ± 6.3 *I* = 32.1 ± 6.2 *Gestational age at recruitment* 16–20 weeks *Duration of study* until 34–36 weeks of gestation	Standard prenatal care	*Nutrition* Individualized nutrition plan with total energy intake of approx 2000 kcal/d 40%–55% of total energy intake from CHO *Physical activity* Supervised exercise program *Frequency* 3-4/week *Intensity* 30% heart rate reserve *Duration* 25–40 min *Type * walking Delivered by dieticians and kinesiologists	NA	6.8 ± 4.1* *Adherence to 1990 IOM* 80% *GWG during the intervention	— —	NA	*At 8 w PP* 2.2 ± 5.6 53% within 2.0 kg of pre-pregnancy body weight	—	3,590 ± 500 <2,500 g OW 4% OB 3% 4,000–4,500 g OW 18% OB 13.3%	3,564 ± 600 <2,500 g OW 0% OB 0% 4,000– 4,500 g OW 3.2% OB 26%	NS NS NS **0.05** 0.07

Guelinckx et al. [65] Design RCT *Country * Belgium *Ethnicity* Caucasians *Prepreg BMI* >29 *Duration of study* until 32 weeks of gestation											

*C* = 43 PI = 37 *Age* *C* = 29.4 ± 4.4 PI = 28.7 ± 4.0 *Parity* *C* = 40% nulliparous PI = 41% nulliparous *SES* *C* = na PI = na *Gestational age at recruitment * *C* = 10.2 ± 2.4 PI = 10.2 ± 2.6	Standard prenatal care	*Passive Intervention:* Received a brochure designed for the study providing advice about nutrition, physical activity and tips to limit GWG	*At delivery* 10.6 ± 6.9 *Adherence to 1990 IOM* 52% did not exceed	*At delivery* 10.9 ± 5.6 *Adherence to 1990 IOM* 54% did not exceed	NS NS	NA	NA	—	3,419 ± 425 >4,000 g *n* = 3 (7%)	3,585 ± 398 >4,000 g *n* = 5 (14%)	NS NS

*C* = 43 AI = 42 *Age* *C* = 29.4 ± 4.4 AI = 28.0 ± 3.6 *Parity* *C* = 40% nulliparous AI = 48% nulliparous *SES* *C* = na AI = na *Gestational age at recruitment* *C* = 10.2 ± 2.4 AI = 9.3 ± 2.8	Standard prenatal care	*Active Intervention:* Received the same brochure plus received 3 counseling group-sessions given by a dietician Delivered by dieticians	*At delivery* 10.6 ± 6.9 *Adherence to 1990 IOM* 52% did not exceed	*At delivery* 9.8 ± 7.6 *Adherence to 1990 IOM* 59% did not exceed	NS NS	NA	NA	—	3,419 ± 425 >4,000 g *n* = 3 (7%)	3,492 ± 468 >4,000 g *n* = 5 (12%)	NS NS

Phelan et al. [59] Design RCT *N* = 401 *C* = 200 *I* = 201 *Country* United States *Ethnicity* *C* = 68% Caucasian *I* = 69% Caucasian *Age* *C* = 28.8 ± 5.2 *I* = 28.6 ± 5.2 *Parity* *C* = 77% nulliparous *I* = 76% nulliparous *SES* *C* = 19% unemployed *I* = 16% unemployed *Prepreg BMI* *C* = 26.5 ± 5.9 *I* = 26.3 ± 5.6	Standard prenatal care	One face-to-face visit at study entry to discuss *Nutrition* Recommendations appropriate calorie goals (20 kcal/kg). *Physical activity* Recommendations appropriate PA (30 min walking most days of the week). *Other* Recommendations appropriate GWG. Food records, pedometers and body weight scale were provided, postcard about healthy eating and exercise habits	*At final visit * NW 16.2 ± 4.6 OW 15.1 ± 7.5 *Adherence to 1990 IOM* NW 48% OW 39% did not exceed	*At final visit* NW 15.3 ± 4.4 OW 14.7 ± 6.9 *Adherence to 1990 IOM* NW 60% OW 33% did not exceed	>0.05 >0.05 **0.003** NS	*At 6 months* NW 3.3 ± 3.5 OW 4.3 ± 6.2 at or below pre-pregnancy body weight NW 21% OW 17%	*At 6 months* NW 2.1 ± 4.7 OW 3.7 ± 5.9 at or below pre-pregnancy body weight NW 36% OW 26%	OR = 2.1 *P* = **0.005**	NW 3,271 ± 467 OW 3,442 ± 629 <2,500 g NW = 5 (5%) OW = 4 (5%) >4,000 g NW = 3 (3%) OW = 14 (16%)	NW 3,367 ± 459 OW 3,430 ± 650 <2,500 g NW = 4 (4%) OW = 5 (6%) >4,000 g NW = 6 (7%) OW = 14 (17%)	NS NS NS NS NS NS
*Gestational age at recruitment* 10–16 weeks *Duration of study* NA		were mailed weekly. 3 brief phone calls from the dietician Personalized graph of GWG Delivered by dieticians									

**Table:** (b) Prenatal intervention studies using the 2009 IOM gestational weight gain recommendations

Reference, Sample characteristics	Treatment		Gestational weight gain (GWG) (kg)			Maternal weight retention postpartum (PP) (kg)			Infant birth weight (g)		
	Control	Intervention	Control	Intervention	*P* value	Control	Intervention	*P* value	Control	Intervention	*P* value
Claesson et al. [56] Design non RCT *N* = 368 *C* = 208 *I* = 160 *Country* Sweden *Ethnicity* Swedish *Age* *C* = 30.2 ± 4.9 *I* = 29.7 ± 4.5 *Parity* *C* = 48% nulliparous *I* = 42% nulliparous *SES* *C* = 26% unskilled workers *I* = 20% unskilled workers *Prepreg BMI* BMI ≥ 30 *Gestational age at recruitment* 10–12 weeks *Duration of study* NA	Standard prenatal care	Motivational interview/talk in early pregnancy *Nutrition* Information about nutrition during pregnancy *Physical activity* Possibility to attend aqua aerobic classes (1-2/week) *Other* 30 min-visit/week for motivational talks, weight control, Additional written information about eating and food intake if needed Delivered by trained midwives	*At delivery* 11.3 ± 5.8 *<7.0 kg* *(study goal)* 21%	*At delivery* 8.7 ± 5.5 *<7.0 kg (study goal)* 36%	**<0.001** **0.003**	*At 10–12 w PP* 0.75 ± 5.34	*At 10–12 w PP* −2.15 ± 5.88	**<0.001**	3,679 ± 571	3,688 ± 681	0.89

Shirazian et al. [57] Design Historical cohort *N* = 28 *C* = 20 *I* = 21 *Country* United States *Ethnicity* *C* = 80% Latina *I* = 67% Latina *Age* *C* = 24.4 ± 5.6 *I* = 29.0 ± 5.1 *Parity* *C* = 50% nulliparous *I* = 19% nulliparous *SES* NA *Prepreg BMI* *C* = 34.2 ± 5.3 *I* = 36.2 ± 5.2 *Gestational age at recruitment* ≤12 weeks *Duration of study* until delivery	Standard prenatal care	6 seminars and at least 5 one-on-one counseling sessions or phone calls *Nutrition* Written material, seminars and counseling sessions to promote healthy eating, facilitate calories counting, kept food diary *Physical activity* Written material, seminars and counseling sessions to encourage walking, received pedometer to monitor *Other* Written material, seminars and counseling sessions to educate the women on obesity and pregnancy and healthy living; goal: GWG < 7 kg Delivered by study coordinators	*Last body weight* 15.4 ± 7.5* ≤*6.8 kg* *(study goal)* 15% did not exceed *Time of last body weight measurement unclear	*Last body weight* 8.1 ± 7.4* ≤*6.8 kg* *(study goal)* 38% did not exceed *Time of last body weight measurement unclear	**0.003** 0.16	NA	NA	—	3,610 ± 548	3,458 ± 603	0.40

Lindholm et al. [66] Design single-arm study *N* = 25 *Country* Sweden *Ethnicity* Swedish *Age* 31.7 ± 3.2 *Parity* median = 1 (0–3) *SES* na *Prepreg BMI* 35.4 ± 4.4 *Gestational age at recruitment* 8–12 weeks *Duration of study* NA		Meeting a midwife every 15 days and 2 group support sessions. *Nutrition* Initial individual consultation, instructed to eat after “the plate model” and to keep an all-inclusive food intake diary. *Physical activity* Supervised exercise program *Frequency* 1/week *Intensity* NA *Duration* 40 min *Type* Water aerobics Asked to exercise 30 min/day PA on other days Delivered by midwives and dietician	NA	*Last body weight* 6.9 ± 6.4* ≤6.0 kg (goal of study) 56% *Time of last body weight measurement unclear	— —	NA	NA	—	NA	3,484 ± 453	—

Hui et al. [60] Design RCT *N* = 190 *C* = 88 *I* = 102 *Country* Canada *Ethnicity* *C* = 25% aboriginal *I* = 17% aboriginal *Age* *C* = 28.7 ± 5.9 *I* = 30.1 ± 5.2 *Parity* *C* = na *I* = na *SES* *C* = 48,602 ± 29,628 (high) *I* = 50,833 ± 23,792 (high) *Prepreg BMI* *C* = 25.7 ± 5.1 *I* = 24.9 ± 5.4 *Gestational age at recruitment* <26 weeks *Duration of study* until 36 weeks of gestation	Standard prenatal care	*Nutrition* Personalized dietary counseling *Physical activity* Supervised exercise session *Frequency* 1/week *Intensity* mild-to-moderate *Duration* 30–45 min *Type * aerobic and strength exercise Women were asked to perform several sessions of exercise sessions at home. VHS and DVD to assist with home-based exercises Delivered by dietitians and licensed fitness trainers	At delivery 15.2 ± 5.9 Adherence to 2009 IOM 45% did not exceed	At delivery 14.1 ± 6.0 *Adherence to 2009 IOM* 65% did not exceed	0.28 **0.008**	NA	NA	—	3,516 ± 530 LGA *n* = 15 (17%)	3,490 ± 509 LGA *n* = 12 (12%)	0.73 0.41

Vinter et al. [62] Design RCT *N* = 304 *C* = 154 *I* = 150 *Country* Denmark *Ethnicity:* Danish *Age:* *C* = 29.0 (27–32) *I* = 29.0 (26–31) *Parity* *C* = 55% nulliparous *I* = 53% nulliparous *SES* *C* = 65% ≥12 yrs *I* = 74% ≥12 yrs *Prepreg BMI:* *C* = 33.3 (31.7–36.9) *I* = 33.4 (30.7–36.5) *Gestational age at recruitment* 10–14 weeks *Duration of study* until delivery	Access to a website with nutrition and PA advices	*Nutrition* Personalized dietary counseling 4 times during the intervention *Physical activity* free membership to gym Supervised exercise session 1x week *Frequency* 1/week *Intensity* moderate *Duration*60 min *Type* aerobic, elastic band, balance exercise *Other* group sessions 4–6 times. Women were encouraged to be active every day for 30–60 min Pedometers were provided Delivered by dieticians and physiotherapists	*At 35 weeks gestation* 8.6 (5.7–11.5) *Adherence to 2009 IOM* 53% did not exceed	*At 35 weeks gestation* 7.0 (4.7–10.6) *Adherence to 2009 IOM* 65% did not exceed	**0.01** 0.058	NA	NA	—	3,593 (3,335–3,930) >4,000 g *n* = 39 (25%)	3,742 (3,464–4,070) >4,000 g *n* = 40 (32%)	**0.039** 0.07

Abbreviations: RCT: randomized controlled trial; *N*: total number; *C*: control group; *I*: intervention group; SES: socioeconomic status; prepreg BMI: body mass index prior to pregnancy; IOM: Institute of Medicine; NA: non available; NS: non significant, NW: normal weight; OW: overweight; OB: obese.

**Table:** (a) Prenatal intervention studies using the 1990 IOM gestational weight gain recommendations

Reference, Sample characteristics	Treatment		Gestational weight gain (GWG) (kg)			Maternal weight retention postpartum (PP) (kg)			Infant birth weight (g)		
	Control	Intervention	Control	Intervention	*P* value	Control	Intervention	*P* value	Control	Intervention	*P* value
Yeo [71] Design RT Stretching (S) versus walking (W) *N* = 124 *S* = 60 *W* = 64 *Country* United States *Ethnicity* *S* = 82% Caucasians *W* = 87% Caucasians *Age* 31.0 ± 5.0 *Parity* NA *SES* *S* = 43% >75,000 (high) *W* = 61% >75,000 (high) *Prepreg BMI* *S* = 98% ≥26 kg/m^2^ *W* = 97% ≥26 kg/m^2^ *Gestational age at recruitment* <14 weeks *Duration of study* until end of pregnancy	—	*Physical activity:* Supervised and home-based exercise program *Stretching: Frequency* 5/week *Intensity* — *Duration* 40 min *Type* Videotape *Walking: * *Frequency * 5/week *Intensity * 55–69% of HR max *Duration * 40 min Delivered by exercise specialists	*Stretchers At delivery* 15.9 ± 6.8 *Adherence to 1990 IOM* 11% did not exceed	*Walkers At delivery* 15.4 ± 5.9 *Adherence to 1990 IOM* 0% did not exceed	NS NS	NA	NA	—	NA	NA	—

**Table:** (b) Prenatal intervention studies using the 2009 IOM gestational weight gain recommendations

Reference, Sample characteristics	Treatment		Gestational weight gain (GWG) (kg)			Maternal weight retention postpartum (PP) (kg)			Infant birth weight (g)		
	Control	Intervention	Control	Intervention	*P* value	Control	Intervention	*P* value	Control	Intervention	*P* value
Cavalcante et al. [70] Design RCT *N* = 71 *C* = 37 *I* = 24 *Country* Brazil *Ethnicity* NA *Age* NA *Parity* NA *SES* Low SES *Prepreg BMI* *C* = 23.4 ± 3.8 *I* = 24.1 ± 4.5 *Gestational age at recruitment* 18–20 weeks *Duration of study* until end of pregnancy	Sedentary during pregnancy	*Physical activity:* Supervised exercise program *Frequency* 3/week *Intensity* 70% pred HR max *Duration* 50 min *Type* Water aerobic Delivered by trained instructors	*At delivery* 15.1 ± 1.6 *Adherence to IOM * NA	*At delivery* 14.3 ± 2.1 *Adherence to IOM* NA	0.38	NA	NA	—	3,313 ± 656 <2,500 g *n* = 2 (37%)	3,222 ± 563 <2,500 g *n* = 3 (33%)	0.54 0.44

Barakat et al. [69] Design RCT *N* = 160 *C* = 80 *I* = 80 *Country* Spain *Ethnicity:* NA *Age:* *C* = 29.5 ± 3.7 *I* = 30.4 ± 2.9 *Parity* *C* = 57% nulliparous *I* = 72% nulliparous *SES* *C* = 13% high school *I* = 26% high school *Prepreg BMI:* *C* = 23.4 ± 0.5 *I* = 24.3 ± 0.5 *Gestational age at recruitment* 12-13 weeks *Duration of study* until end of pregnancy	Continue usual daily activities	*Physical activity:* Supervised exercise program *Frequency* 3/week *Intensit*y 60% HR max *Duration* 35–40 min *Type* Muscle strengthening and toning program	*At delivery* 12.4 ± 3.4 *Adherence to 2009 IOM* NA	*At delivery* 11.5 ± 3.7 *Adherence to 2009 IOM* NA	>0.1	NA	NA	—	3,307 ± 477 <2,500 g *n* = 4 (6%) >4,000 g *n* = 7 (10%)	3,165 ± 411 <2,500 g *n* = 4 (6%) >4,000 g *n* = 1 (1%)	>0.1 >0.1 >0.1

Barakat et al. [72] Design RCT *N* = 83 *C* = 43 *I* = 40 *Country* Spain *Ethnicity* NA *Age* *C* = 31 ± 3 *I* = 32 ± 4 *Parity* *C* = 49% nulliparous *I* = 65% nulliparous *SES* *C* = 14% >high school *I* = 83% >high school *Prepreg BMI:* *C* = 23.0 ± 2.9 *I* = 22.7 ± 2.8 *Gestational age at recruitment* 6–9 weeks *Duration of study* until end of pregnancy	Continue usual daily activities	*Physical activity:* Supervised exercise program *Frequency* 3/week *Intensit*y ≤70% HR max *Duration* 35–45 min *Type* aerobic class (2/week) aquatic activities (1/week)	*At delivery* 13.8 ± 3.1 *Adherence to 2009 IOM* NA	*At delivery* 12.5 ± 3.2 *Adherence to 2009 IOM* NA	>0.05	NA	NA	—	3,465 ± 411	3,404 ± 465	>0.05

Haakstad and Bø [67] Design RCT *N* = 105 *C* = 53 *I* = 52 *Country* Norway *Ethnicity* NA *Age* *C* = 30.3 ± 4.4 *I* = 31.2 ± 3.7 *Parity* 100% nulliparous *SES* *C* = 85% college/university *I* = 85% college/university *Prepreg BMI* *C* = 23.9 ± 4.7 *I* = 23.8 ± 3.8 *Gestational age at recruitment* *C* = 18.0 ± 4.3 *I* = 17.3 ± 4.1 Duration of study 12 weeks	Continue usual daily activities	*Physical activity:* Supervised exercise program *Frequency* ≥2/week *Intensity* 12–14 RPE *Duration* 60 min *Type* Aerobic dance Women were asked to exercise 30 min/day on other days Delivered by certified aerobic instructors	*At 36–38 weeks* 13.8 ± 4.0 *Adherence to 2009 IOM * 62% did not exceed	*At 36–38 weeks* 13.0 ± 4.0 If attendance to all ex sessions (2/week, *n* = 24) 11.0 ± 2.0 *Adherence to 2009 IOM* 67% if ex 2/week 0% did not exceed	0.31 **0.01** 0.59 **0.006**	*At 8.1 ± 1.5 w PP* 3.3 ± 4.1	*At 7.1 ± 1.6* *we PP* 3.3 ± 3.9 if ex sessions 2/week 0.8 ± 1.7	0.93 **0.001**	NA	NA	—

Nascimento et al. [68] Design RCT *N* = 82 *C* = 42 *I*= 40 *Country* Brazil *Ethnicity* NA *Age* *C* = 30.9 ± 5.9 *I* = 29.7 ± 6.8 *Parity* *C* = 24% nulliparous *I* = 30% nulliparous *SES* *C* = 38% >12 yr of school *I* = 43% >12 yr of school *Prepreg BMI* *C* = 36.4 ± 6.9 *I* = 34.8 ± 6.6 *Gestational age at recruitment * *C* = 17.8 ± 3.7 *I* = 17.6 ± 4.2 *Duration of study* until end of pregnancy	Standard prenatal care	*Physical activity:* Supervised exercise program *Frequency* 1/week *Intensity* HR < 140 *Duration* 40 min *Type* Aerobic dance Women were asked to exercise 5/week at home *Other:* women were counseled on recommended GWG Delivered by trained physical therapists	*At final visit* 11.5 ± 7.4 OB = 10.9 ± 7.6 OW =16.4 ± 3.9 *Adherence to 2009 IOM* 43% did not exceed	*At final visit* 10.3 ± 5.0 OB = 10.4 ± 5.6 OW = 10.0 ± 1.7 *Adherence to 2009 IOM* 52% did not exceed	0.54 0.76 **0.001** 0.43	NA	NA	—	3,228 ± 591 SGA *n* = 1 (3%) LGA *n* = 8 (24%)	3,267 ± 700 SGA *n* = 2 (6%) LGA *n* = 8 (24%)	0.79 NS NS

Abbreviations: RCT: randomized controlled trial; *N*: total number; *C*: control group; *I*: intervention group; SES: socioeconomic status; prepreg BMI: body mass index prior to pregnancy; IOM: Institute of Medicine; NA: non available; NS: non significant, NW: normal weight; OW: overweight; OB: obese.

**Table 4:** Summary of the effects of prenatal lifestyle intervention studies with regard to gestational weight gain, maternal weight retention, and infant birth weight.

Reference	Intervention	Gestational weight gain (GWG)		Maternal weight retention postpartum	Changes in infant birth weight/prevention of large and small infant at birth	
Gray-Donald et al. [64]	Nutrition (recommendations) Physical activity (supervised) Advice to stay within GWG recommendations	*Mean GWG* unsuccessful	*Adherence to 1990 IOM * NA	*Mean weight retention at 6 weeks* unsuccessful	*Mean birth weight * unsuccessful	

Polley et al. [58]	Nutrition (newsletters) Physical activity (newsletters) Info about appropriate GWG	*Mean GWG* unsuccessful	*Adherence to 1990 IOM* **Partially successful (only in NW women)**	*Mean weight retention at 8 ± 7 weeks* unsuccessful	*Mean birth weight * unsuccessful	

Olson et al. [61]	Nutrition (newsletters) Physical activity (newsletters) Guidance about self-monitoring of GWG	*Mean GWG* unsuccessful	*Adherence to 1990 IOM* **Partially successful (only in low SES women)**	*Mean weight retention at 1 year* **Partially successful (only in low SES OW women)**	*Mean birth weight * unsuccessful	

Kinnunen et al. [63]	Nutrition (counseling) Physical activity (supervised) Guidance about self-monitoring of GWG	*Mean GWG* unsuccessful	*Adherence to 1990 IOM* unsuccessful	*Mean weight retention* NA	*Mean birth weight * NA	*≥4,000 g* **successful**

Asbee et al. [55]	Nutrition (plan) Physical activity (recommendations) Information about GWG guidelines	*Mean GWG* **successful **	*Adherence to 1990 IOM* unsuccessful	*Mean weight retention* NA	*Mean birth weight * NA	

Mottola et al. [44]	Nutrition (plan) Physical activity (supervised)	*Mean GWG* **successful**	*Adherence to 1990 IOM* **successful**	*Mean weight retention at 8 weeks* **successful**	*Mean birth weight * unsuccessful	*<2,500 g* **successful** *4,000–4,500 g* **successful**

Guelinckx et al. [65]	Nutrition (brochure/counseling) Physical activity (brochure/counseling) Tips to limit GWG	*Mean GWG* unsuccessful	*Adherence to 1990 IOM* unsuccessful	*Mean weight retention* NA	*Mean birth weight * unsuccessful	*>4,000 g* unsuccessful

Phelan et al. [59]	Nutrition (recommendations) Physical activity (recommendations) Recommendations about appropriate GWG	*Mean GWG* unsuccessful	*Adherence to 1990 IOM* **Partially successful (only in NW women)**	≤*pre-pregnancy body weight at 6 months* **successful**	*Mean birth weight * unsuccessful	*<2,500 g* unsuccessful *>4,000 g* unsuccessful

Claesson et al. [56]	Nutrition (recommendations) Physical activity (supervised) Motivational interview	*Mean GWG* **successful**	*<7.0 kg (study goal) * **successful**	*Mean weight retention at 10–12 weeks* **successful**	*Mean birth weight * unsuccessful	

Shirazian et al. [57]	Nutrition (counseling) Physical activity (counseling) GWG goal	*Mean GWG* **successful**	≤*6.8 kg (study goal)* unsuccessful	*Mean weight retention* NA	*Mean birth weight* unsuccessful	

Lindholm et al. [66]	Nutrition (counseling) Physical activity (supervised)	*Mean GWG *	≤*6.0 kg (goal of study) * unsuccessful	*Mean weight retention* NA	*Mean birth weight * NA	

Hui et al. [60]	Nutrition (counseling) Physical activity (supervised)	*Mean GWG* unsuccessful	*Adherence to 2009 IOM* **successful**	*Mean weight retention* NA	*Mean birth weight * unsuccessful	*LGA (not defined)* unsuccessful

Vinter et al. [62]	Nutrition (counseling) Physical activity (supervised)	*Mean GWG* **successful**	*Adherence to 2009 IOM* **successful (*P* = 0.058)**	*Mean weight retention* NA	*Mean birth weight * unsuccessful	*>4,000 g* unsuccessful

Yeo [71]	Physical activity (supervised)	*Mean GWG* unsuccessful	*Adherence to 1990 IOM* unsuccessful	*Mean weight retention* NA	*Mean birth weight * NA	

Cavalcante et al. [70]	Physical activity (supervised)	*Mean GWG* unsuccessful	*Adherence to IOM * NA	*Mean weight retention* NA	*Mean birth weight * unsuccessful	*<2,500 g* unsuccessful

Barakat et al. [69]	Physical activity (supervised)	*Mean GWG* unsuccessful	*Adherence to 2009 IOM * NA	*Mean weight retention* NA	*Mean birth weight * unsuccessful	*<2,500 g* unsuccessful *>4,000* unsuccessful

Barakat et al. [72]	Physical activity (supervised)	*Mean GWG* unsuccessful	*Adherence to 2009 IOM * NA	*Mean weight retention* NA	*Mean birth weight * unsuccessful	

Haakstad and Bø [67]	Physical activity (supervised)	*Mean GWG* **Partially successful** **(only if attendance to 2 ex sessions/week)**	*Adherence to 2009 IOM* **Partially successful ** **(only if attendance to 2 ex sessions/week)**	*Mean weight retention at 8–12 weeks* **Partially successful** **(only if attendance to 2 ex sessions/week)**	*Mean birth weight * NA	

Nascimento et al. [68]	Physical activity (supervised)	*Mean GWG* **Partially successful** **(in OW women)**	*Adherence to 2009 IOM* unsuccessful	*Mean weight retention* NA	*Mean birth weight * unsuccessful	*SGA (not defined)* unsuccessful *LGA * *(not defined)* unsuccessful

Abbreviations: IOM: Institute of Medicine; NA: non available: SES: socioeconomic status; NW: normal weight; OW: overweight.
